# 

**Published:** 1864-03

**Authors:** 


### CONTENTS.

### ORIGINAL CONTRIBUTIONS.

ART. IX. About Pocket Surgical Cases. By E. Andrews, M.D., Professor of Surgery in Chicago Medical College,........................... 129
ART. X. Catalysis as a Chemical, Physiological, and Pathological Process. By John Reid, M.D., of Chicago, Ill. (Communicated to the Chicago Medical Society,).......................... 132
ART. XI. Animal Poisons as Causes of Diseases; Indications for Treatment in Diseases Produced by them. By N. S. Davis, M.D., Professor of Practical and Clinical Medicine. (Remarks made in a Discussion before the Chicago Medical Society,) .................. 144
ART. XII. Sanitary Report of the West Division of the City of Chicago. By Hiram Wanzer, M.D., of Chicago, Ill. (Read before the Chicago Medical Society, Feb. 26, 1864,)...................... 149

### SELECTIONS.

On the Therapeutical Applications of the Solution of the Permanganate of Potash, and of Ozone. By Samuel Jackson, M.D., Emeritus Prof. of the Institutes of Medicine in the University of Pennsylvania, ... 154
On the Constitution and Source of the Bile. By Thomas Antisell, Surgeon U.S.V., Professor of Physiology and Military Surgery in the Medical Department of Georgetown College, D.C.,....................... 162
The French System of Medical Education,.................................. 171
Mortality of Liverpool,.................................................. 178
British Surgery in Paris,................................................ 178

### BOOK NOTICES.

The Medical Formulary: Being a Collection of Prescriptions, &c. By R. P. Thomas, M.D., &c. Blanchard & Lea, Philadelphia,.................. 179
On Asthma: Its Pathology and Treatment. By Henry H. Salter, M.D., &c. Blanchard & Lea, Philadelphia,.................................... 179
Braithwaite's Retrospect of Practical Medicine and Surgery—for the half-year ending January, 1864. W. A. Townsend, New York,............. 180
To Know: Its Source, its Mode, and its Power. By Frank W. White, A.M., M.D.,................................................................ 181
A Treatise on Practical Pharmacy. By Edward Parrish. Blanchard & Lea, Philadelphia,.................................................... 181

### EDITORIAL.

Illinois State Medical Society,.......................................... 181
Chicago Medical College,................................................. 182
Letter from Dr. Henry Wing,............................................. 183
American Medical Association,............................................ 183
Letter from Dr. W. N. Cote,.............................................. 184
Formula for a Solution of Bromine,....................................... 188
On the Use of Collodion with Glycerine in Erysipelas,.................... 189
Professional Dignity in France,.......................................... 189
Perseverence,............................................................ 189

